# Supplementary material for: Regional patterns of human cortex development correlate with underlying neurobiology
Source: Nat Commun. 2024 Sep 12;15:7987. doi: 10.1038/s41467-024-52366-7 (PMC11405413; doi:10.1038/s41467-024-52366-7)
Supplement: Supplementary file 6 — Reporting Summary [file 41467_2024_52366_MOESM6_ESM.pdf]

nature portfolio

Corresponding author(s): Leon D. Lotter; Juergen Dukart

Last updated by author(s): Jul 17, 2024

Reporting Summary

Nature Portfolio wishes to improve the reproducibility of the work that we publish. This form provides structure for consistency and transparency in reporting. For further information on Nature Portfolio policies, see our Editorial Policies and the Editorial Policy Checklist.

Statistics

For all statistical analyses, confirm that the following items are present in the figure legend, table legend, main text, or Methods section.

n/a	Confirmed	
☐	☒	The exact sample size (n) for each experimental group/condition, given as a discrete number and unit of measurement
☐	☒	A statement on whether measurements were taken from distinct samples or whether the same sample was measured repeatedly
☐	☒	The statistical test(s) used AND whether they are one- or two-sided<br>*Only common tests should be described solely by name; describe more complex techniques in the Methods section.*
☐	☒	A description of all covariates tested
☐	☒	A description of any assumptions or corrections, such as tests of normality and adjustment for multiple comparisons
☐	☒	A full description of the statistical parameters including central tendency (e.g. means) or other basic estimates (e.g. regression coefficient) AND variation (e.g. standard deviation) or associated estimates of uncertainty (e.g. confidence intervals)
☐	☒	For null hypothesis testing, the test statistic (e.g. F, t, r) with confidence intervals, effect sizes, degrees of freedom and P value noted<br>*Give P values as exact values whenever suitable.*
☒	☐	For Bayesian analysis, information on the choice of priors and Markov chain Monte Carlo settings
☒	☐	For hierarchical and complex designs, identification of the appropriate level for tests and full reporting of outcomes
☐	☒	Estimates of effect sizes (e.g. Cohen's d, Pearson's r), indicating how they were calculated

Our web collection on statistics for biologists contains articles on many of the points above.

Software and code

Policy information about availability of computer code

Data collection	No software was used in data collection.
Data analysis	All analyses were conducted in a Python (3.9.11) environment using openly available toolboxes and custom code available at https://github.com/LeonDLotter/CTdev (DOI: 10.5281/zenodo.7902901). The core analyses aiming to draw associations between cortical thickness distributions and multimodal brain atlases were conducted using JuSpyce 0.0.2 available at https://github.com/LeonDLotter/JuSpyce. Multimodal brain atlases were retrieved and processed from/with neuromaps (0.0.2), abagen (0.1.3), JuSpace (1.3), or author sources. Analyses of associations between CT and cortical atlases were conducted using JuSpyce 0.0.2 in a Python 3.9.11 environment. JuSpyce (https://github.com/LeonDLotter/JuSpyce) is a toolbox allowing for flexible assessment and significance testing of associations between multimodal neuroimaging data, relying on imaging space transformations from neuromaps, brain surrogate map generation from BrainSMASH (0.11.0), and several routines from Nilearn (0.10.2), scipy (1.12.0), NiMARE (0.0.11), statsmodels (0.14.1), pingouin (0.5.4), numpy (1.22.4), and pandas (1.5.3). Visualizations were created using matplotlib (3.8.3), seaborn (0.11.0), and surfplot (0.1.0). The PCNtoolkit (0.29.post1) was used to generate modeled CT data, as well as predicted CT data and deviation scores for ABCD and IMAGEN subjects; neuroHarmonize (2.3.1) was used to independently harmonize the CT data across sites.

For manuscripts utilizing custom algorithms or software that are central to the research but not yet described in published literature, software must be made available to editors and reviewers. We strongly encourage code deposition in a community repository (e.g. GitHub). See the Nature Portfolio guidelines for submitting code & software for further information.

Data

Policy information about availability of data

All manuscripts must include a data availability statement. This statement should provide the following information, where applicable:

- Accession codes, unique identifiers, or web links for publicly available datasets
- A description of any restrictions on data availability
- For clinical datasets or third party data, please ensure that the statement adheres to our policy

Neurobiological marker source data, the Braincharts models, data extracted from these models, developmental gene expression data, and colocalization results are available from a GitHub repository accompanying this publication (https://github.com/LeonDLotter/CTdev/; DOI: 10.5281/zenodo.7902901). The Braincharts model is furthermore available from: https://github.com/predictive-clinical-neuroscience/braincharts. Original data and derivatives from the ABCD and IMAGEN datasets cannot be shared openly but are accessible via requests to the original investigators (https://abcdstudy.org/; https://imagen-project.org/).

Research involving human participants, their data, or biological material

Policy information about studies with human participants or human data. See also policy information about sex, gender (identity/presentation), and sexual orientation and race, ethnicity and racism.

Reporting on sex and gender	The main cortical thickness (CT) association results were based on reference CT data extracted from the "Braincharts" normative model by Rutherford et al. (2022). The Braincharts model was estimated including sex and age as covariates and therefore allows to predict CT for a given age and (binary) sex. We extracted 50th percentile CT data across the lifespan for both males and females and assessed CT (change) associations separately by sex. As we found that the association patterns were highly similar between sexes (see Figs. S8–S10), we averaged the CT data across sexes in all model-based analyses reported in the main results.<br>The single-subject analyses aimed to validate the CT association patterns observed in modeled CT data. Therefore, we reported the results independent of sex in the main manuscript. In the supplementary materials, we reported on sex-differences concerning the extent to which CT change patterns were explained (see Fig. S26).<br>Included multilevel brain atlases were derived from different populations with varying sex distributions (see Tab. S1).
Reporting on race, ethnicity, or other socially relevant groupings	The Braincharts model was trained on a large part of the MRI neuroimaging data available to date. This data is biased in the sense that it mostly includes subjects of Central European origin. We clearly mention this limitation of the model and thus of our findings in the manuscript. For ABCD and IMAGEN data, no consistent assessment of ethnicity was available. However, we report on differences across study sites concerning brain-regional CT, CT change, and the the extent to which CT change patterns were explained (see Tab. S2–S3 and Fig. S26). Because the ethnicity of subjects may vary considerably by study site, site differences could in part reflect effects of ethnicity, which remains a subject for further study.
Population characteristics	Braincharts model: n = 58,836, 82 sites, 51% female, 50% training/testing split. The age distribution is shown in Fig. S5. Information about study sites is available from Rutherford et al. (2022).<br>ABCD: n = 6,789, 21 sites, 2 time points, 8.9 – 13.8 years. Detailed information in Tab. S2 and from Casey et al. (2018).<br>IMAGEN: n = 1,326, 8 sites, 3 time points, 13.3 – 25.1 years. Detailed information in Tab. S2 and from Schumann et al. (2010).<br>Multilevel brain atlases: n = 6 – 417, mean age 19.9 – 66.8 years. Detailed information in Tab. S1.
Recruitment	All analyses in this manuscript were based on existing data. Recruitment for each dataset was described in Rutherford et al. (2022) for the Braincharts model, Casey et al. (2018) for the ABCD dataset, Schumann et al. (2010) for the IMAGEN data, and in each original publication associated to the multilevel brain atlases (see Tab. S1).
Ethics oversight	No new human data were acquired for this study. Ethical approval for usage of publicly available and restricted-access databanks including human demographic, behavioral, and neuroim-aging data has been granted by the Heinrich-Heine-University, Düsseldorf, Germany. Specific ap-proval for collection and sharing of the used data (brain atlases, Braincharts model, Human Brain Transcriptome, ABCD, and IMAGEN) were provided by local ethics committees; detailed infor-mation is available in the cited sources. Use of the ABCD data is registered at the NDA database at http://dx.doi.org/10.15154/1528657. The responsible IMAGEN investigator is T. Banaschewski.

Note that full information on the approval of the study protocol must also be provided in the manuscript.

Field-specific reporting

Please select the one below that is the best fit for your research. If you are not sure, read the appropriate sections before making your selection.

☒ Life sciences ☐ Behavioural & social sciences ☐ Ecological, evolutionary & environmental sciences

For a reference copy of the document with all sections, see nature.com/documents/nr-reporting-summary-flat.pdf

Life sciences study design

All studies must disclose on these points even when the disclosure is negative.

Sample size	No a-priori sample size calculation was performed. The Braincharts model was built on the majority of neuroimaging data available to date and thoroughly validated by Rutherford et al. (2022). The ABCD and IMAGEN datasets constitute two of the largest published longitudinal

developmental MRI datasets. We conducted sensitivity analyses and visualized whole-sample distributions where possible to demonstrate the robustness of our results.

Data exclusions	Quality of FreeSurfer CT reconstruction was determined using cohort-specific thresholds set on Euler number-related metrics (i.e., the number of surface defects). Subjects were excluded based on these metrics and the quality ratings provided in the ABCD dataset. Only ABCD and IMAGEN subjects with at least two valid measurement time points were included in the main analyses. The numbers of excluded subjects were reported in the Supplementary Materials section 1.4.1.<br>Subjects were excluded from the original Braincharts cohort using a similar approach, which was reported in Rutherford et al. (2022).
Replication	Our main findings were based on the 50th percentile modeled CT data extracted from the Braincharts model. The observed patterns between adolescence and young adulthood, i.e., associations between multilevel brain systems and cross-sectional CT as well as lifespan CT changes, were replicated in the ABCD and IMAGEN datasets. This was done on the cohort level and the study-site level within each cohort (based on the average CT or CT change across each cohort's available time spans), as well as on the individual level (based on the CT or CT change observed for each subject). As described in the manuscript, the patterns detected with the Braincharts modeled data replicated very clearly in ABCD and IMAGEN cohort-average CT change data. On the individual level (ABCD and IMAGEN single-subject CT change from one study time point to another), however, we could observe a very strong inter-individual variability. While the overall pattern replicated here as well, future research will have to investigate potential sources for this variability. Preliminary analyses reported in our manuscript suggest a part of the inter-individual variance to be explained by study site/ scanner and participant's self-reported sex, even after site-harmonization. The question whether potential demographic or ethnic factors play into this finding goes beyond the scope of the present study, warranting a careful and targeted independent investigation.
Randomization	To compare predicted and observed CT data in the individual-subject validation analyses the observed data was projected into the Braincharts model. This process required an adaptation sample used to adjust the model to the effect of sites it was not trained on originally. These adaptation samples were drawn randomly from the ABCD and IMAGEN datasets, while ensuring equal distributions across sites (n = 20 per site), sex (50% male/female), and age. No further randomization of subjects was necessary.<br>Statistical significance of associations between (modeled) CT changes and multilevel brain systems was assessed by permuting each multilevel brain map (10,000 iterations) while retaining spatial autocorrelation patterns.
Blinding	No blinding was possible or desired as all analyses aimed to identify spatial correlates of (modeled) CT change trajectories in a data-driven manner.

Reporting for specific materials, systems and methods

We require information from authors about some types of materials, experimental systems and methods used in many studies. Here, indicate whether each material, system or method listed is relevant to your study. If you are not sure if a list item applies to your research, read the appropriate section before selecting a response.

Materials & experimental systems

n/a	Involved in the study
☒	☐ Antibodies
☒	☐ Eukaryotic cell lines
☒	☐ Palaeontology and archaeology
☒	☐ Animals and other organisms
☒	☐ Clinical data
☒	☐ Dual use research of concern
☒	☐ Plants

Methods

n/a	Involved in the study
☒	☐ ChIP-seq
☒	☐ Flow cytometry
☐	☒ MRI-based neuroimaging

Plants

Seed stocks	Automated hiding of this data field did not work. No plants were used in this work.
Novel plant genotypes	*Describe the methods by which all novel plant genotypes were produced. This includes those generated by transgenic approaches, gene editing, chemical/radiation-based mutagenesis and hybridization. For transgenic lines, describe the transformation method, the number of independent lines analyzed and the generation upon which experiments were performed. For gene-edited lines, describe the editor used, the endogenous sequence targeted for editing, the targeting guide RNA sequence (if applicable) and how the editor was applied.*
Authentication	*Describe any authentication procedures for each seed stock used or novel genotype generated. Describe any experiments used to assess the effect of a mutation and, where applicable, how potential secondary effects (e.g. second site T-DNA insertions, mosiacism, off-target gene editing) were examined.*

Magnetic resonance imaging

Experimental design

Design type	Structural MRI.

Design specifications | No specific experimental setup was applied.

Behavioral performance measures | No behavioral measures were included.

Acquisition

Imaging type(s): T1-weighted structural MRI.

Field strength: ABCD and IMAGEN: 3T; Braincharts: Varying.

Sequence & imaging parameters: Varying (description in each dataset publication).

Area of acquisition: Whole brain.

Diffusion MRI ☐ Used ☒ Not used

Preprocessing

Preprocessing software: FreeSurfer 6.0 (Braincharts) and 7.1.1 (IMAGEN and ABCD)

Normalization: FreeSurfer recon-all.

Normalization template: fsaverage.

Noise and artifact removal: FreeSurfer recon-all.

Volume censoring: None.

Statistical modeling & inference

Model type and settings: Colocalization between cross-sectional (modeled) CT patterns and multilevel brain systems: Spatial Spearman correlations at each tested time point.
"Prediction" of longitudinal (modeled) CT change patterns from multilevel brain systems: Univariate and multivariate regression analyses across each tested time span, extended by dominance analyses to quantify predictor importance (see below).

Effect(s) tested: We evaluated the extent to which multilevel brain systems could explain (modeled) CT change patterns, quantified as the amount of CT change variance explained (adjusted R-squared).

Specify type of analysis: ☒ Whole brain ☐ ROI-based ☐ Both

Statistic type for inference (See Eklund et al. 2016): Not applicable.

Correction: Statistical significance of CT association results was determined using permutation approaches. Benjamini-Hochberg false discovery rate correction was applied to correct for multiple comparisons. The results were reported with a focus on effect sizes to facilitate interpretation.

Models & analysis

n/a	Involved in the study
☒	☐ Functional and/or effective connectivity
☒	☐ Graph analysis
☐	☒ Multivariate modeling or predictive analysis

Multivariate modeling and predictive analysis: Univariate and multivariate linear regression models were used to "predict" (modeled) CT change patterns from multilevel brain systems. The original multilevel atlases were z-standardized and dimensionality reduced using factor analyses, resulting in 21 predictor variables. For the 50th percentile Braincharts data, one model was fitted for each tested timespan using one or multiple multilevel brain maps as predictors and 148 cortex regions as observations to "predict" the modeled CT change pattern across the 148 regions. For the validation cohorts, one model was fitted for either the cohort-average CT change or each subject's individual CT change across a given time span.
Our main objective was to demonstrate how (individual or modeled) CT change patterns could be explained from multilevel brain systems. We thus focused on the CT change variance explained as our main outcome metric rather than on the generalizability of regression models. Where possible, we contrasted the observed results to the amount of CT change variance explained by "null" maps to allow for an assessment of overfitting effects.
